# Quantitative cardiovascular magnetic resonance perfusion imaging identifies reduced flow reserve in microvascular coronary artery disease

**DOI:** 10.1186/s12968-018-0435-1

**Published:** 2018-02-22

**Authors:** Benjamin Zorach, Peter W. Shaw, Jamieson Bourque, Sujith Kuruvilla, Pelbreton C. Balfour, Yang Yang, Roshin Mathew, Jonathan Pan, Jorge A. Gonzalez, Angela M. Taylor, Craig H. Meyer, Frederick H. Epstein, Christopher M. Kramer, Michael Salerno

**Affiliations:** 1 Department of Medicine, Cardiology Division, University of Virginia Health System, Charlottesville, VA USA; 2 Berkshire Medical Center, Pittsfield, MA USA; 3 Department of Radiology, Cardiovascular Imaging Center, University of Virginia Health System, Charlottesville, VA USA; 4 Department of Medicine, Philadelphia VA Medical Center, University of Pennsylvania, Perelman School of Medicine, Philadelphia, PA USA; 5 Department of Biomedical Engineering, University of Virginia Health System, Charlottesville, VA USA; 6 Division of Cardiovascular Disease, Scripps Clinic, Division of Cardiology, Cardiovascular Imaging, Division of Radiology, La Jolla, San Diego, CA USA

**Keywords:** Cardiovascular magnetic resonance, Microvascular disease, Myocardial perfusion reserve, Non-obstructive coronary artery disease, Angina, Women, Diabetes, Metabolic syndrome

## Abstract

**Background:**

Preliminary semi-quantitative cardiovascular magnetic resonance (CMR) perfusion studies have demonstrated reduced myocardial perfusion reserve (MPR) in patients with angina and risk factors for microvascular disease (MVD), however fully quantitative CMR has not been studied. The purpose of this study is to evaluate whether fully quantitative CMR identifies reduced MPR in this population, and to investigate the relationship between epicardial atherosclerosis, left ventricular hypertrophy (LVH), extracellular volume (ECV), and perfusion.

**Methods:**

Forty-six patients with typical angina and risk factors for MVD (females, or males with diabetes or metabolic syndrome) who had no obstructive coronary artery disease by coronary angiography and 20 healthy control subjects underwent regadenoson stress CMR perfusion imaging using a dual-sequence quantitative spiral pulse sequence to quantify MPR. Subjects also underwent T1 mapping to quantify ECV, and computed tomographic (CT) coronary calcium scoring to assess atherosclerosis burden.

**Results:**

In patients with risk factors for MVD, both MPR (2.21 [1.95,2.69] vs. 2.93 [2.763.19], *p* < 0.001) and stress myocardial perfusion (2.65 ± 0.62 ml/min/g, vs. 3.17 ± 0.49 ml/min/g *p* < 0.002) were reduced as compared to controls. These differences remained after adjusting for age, left ventricular (LV) mass, body mass index (BMI), and gender. There were no differences in native T1 or ECV between subjects and controls.

**Conclusions:**

Stress myocardial perfusion and MPR as measured by fully quantitative CMR perfusion imaging are reduced in subjects with risk factors for MVD with no obstructive CAD as compared to healthy controls. Neither myocardial hypertrophy nor fibrosis accounts for these differences.

**Electronic supplementary material:**

The online version of this article (10.1186/s12968-018-0435-1) contains supplementary material, which is available to authorized users.

## Background

Coronary artery disease (CAD) is a major cause of morbidity in the United States. It is estimated that 15.5 million Americans have heart disease and 8.2 million suffer from angina pectoris [1]. In a retrospective analysis of the NCDR Cath Registry, among patients with suspected CAD undergoing elective coronary angiography without known CAD, only 37.6% of patients demonstrated obstructive epicardial CAD [2]. It is now widely recognized that obstructive epicardial CAD is not the sole cause of myocardial ischemia [3].

Indeed, microvascular disease (MVD), or coronary microvascular dysfunction (CMD), is increasingly seen as an important contributor in the pathophysiology of ischemic heart disease and chest pain syndromes [4, 5]. In the WISE study, abnormal coronary microvascular reactivity to adenosine was highly prevalent and a significant predictor of adverse cardiovascular outcomes in women even in the absence of obstructive CAD [6]. In a population of men and women undergoing coronary angiography, women had more cardiovascular events despite less significant obstructive CAD, attributable in part to reduced coronary flow reserve (CFR) [7].

The cause of MVD is thought to be multifactorial likely due impaired vasodilatation of the microcirculation supplying the myocardium, as well as from an increased response to factors that promote vasoconstriction [5]. The risk factors associated with MVD include – but are not limited to – female gender, diabetes, and the metabolic syndrome [8–10]. The cumulative number of risk factors was previously shown to be inversely related to coronary flow reserve [11]. A recent review by a working group in the study of myocardial ischemia and no obstructive coronary artery disease (MINOCA) comprehensively highlights the data behind the current understanding of MVD and proposes future directions of research including detection with noninvasive imaging [12].

The diagnosis of MVD has traditionally been ascertained by means of invasive cardiac catheterization using vasomotor testing with acetylcholine during coronary angiography and/or by CFR during adenosine infusion using invasive coronary Doppler flow measurements [12]. More recently, there has been growing interest in assessing abnormal myocardial perfusion reserve (MPR) in MVD non-invasively using techniques such as positron emission tomography (PET) and cardiovascular magnetic resonance (CMR).

A number of CMR methods have been applied to the measurement of myocardial perfusion for the detection of obstructive CAD, including semi-quantitative and fully quantitative techniques [13, 14]. To date, the efficacy of using fully quantitative CMR to assess MVD has yet to be examined. The aim of the present study is to demonstrate that quantitative CMR identifies reduced MPR in patients with angina and risk factors for MVD who have *no* evidence of obstructive CAD by coronary angiography. A secondary aim of the study was to investigate any relationship between myocardial hypertrophy, inflammation, fibrosis, and epicardial atherosclerosis with myocardial perfusion in this patient population.

## Methods

### Subjects

Patients with risk factors for MVD (women, or men with diabetes mellitus or metabolic syndrome) were prospectively recruited from the University of Virginia Health System. All patients had undergone coronary angiography for evaluation of typical exertional angina and were found to have no obstructive CAD (defined as stenosis > 50% by quantitative coronary angiography in at least one coronary artery or fractional flow reserve < 0.80 in at least one artery and no prior revascularization). Twenty healthy controls without angina and without risk factors for MVD were also recruited as a comparison group. The University of Virginia Health System institutional review board approved this study (Protocol #16056) and all subjects gave written informed consent. An electrocardiogram (ECG) was obtained before the CMR study and resting heart rate and blood pressure were recorded.

### Image acquisition

#### CMR

##### Cine imaging

CMR was performed on a 1.5 T scanner (Avanto or Aera, Siemens Healthineers, Erlangen, Germany) using the standard spine and body phased-array coils. Anatomic and functional assessment of the left ventricle (LV) was performed using a balanced steady-state free precession (bSSFP) cine sequence. The parameters included repetition time (TR) 2.7 ms, echo time (TE) 1.3 ms, flip angle (FA) 73^0^, field of view (FOV) 300-350 mm, and resolution 1.8 × 1.4 × 8.0 mm. A stack of 8 mm thick short axis images with 2 mm gap covered the LV from apex to base. Three long-axis images were obtained (2, 3, and 4-chamber views).

##### Perfusion imaging

For assessment of MPR, quantitative first-pass myocardial perfusion imaging was performed first at rest and then during regadenoson stress following a 15 min contrast washout period. Resting perfusion assessment was performed before regadenoson stress to avoid confounding effects from regadenoson infusion, as it has been previously demonstrated that resting perfusion obtained 20 min after regadenoson with aminophylline reversal was higher than rest perfusion performed before regadenoson [15, 16].

For each perfusion assessment 3 short axis slice locations were imaged per heart beat over a 50 heart beat acquisition during an IV bolus of 0.075 mmol/kg of gadolinium contrast (Magnevist, Bayer Healthcare, Whippany, New Jersey, USA) injected via power injector (Medrad Continuum, Warrendale, Pennsylvania, USA) at 4 cm^3^/s. Images were acquired using an accelerated saturation recovery (SR) variable-density spiral perfusion pulse sequence with integrated proton density (PD) and arterial input function (AIF) acquisitions for perfusion quantification [17–19]. Pulse sequence parameters included: SR time of 80 ms (time to first radiofrequency pulse of the readout), TE 1 ms, TR 9 ms, slice thickness 8 mm, FA 30 degrees, FOV 320 mm, 8 spiral interleaves, 6.1 ms readout duration per spiral and nominal spatial resolution of 1.5 mm^2^. Arterial input function (AIF) images were acquired with a single-shot spiral acquisition with the following parameters: flip angle (FA) 45°, in-plane resolution 6.95mm^2^, SR time 20 ms. The PD images were collected in the first 2 heart beats without a saturation pulse using FA 10° and were used for both quantification and calibration of the SPIRiT kernel for image reconstruction.

Native T1 maps were obtained at base and mid short-axis slice locations prior to the vasodilator stress protocol. T1 maps were obtained at 10 min following the first contrast injection, and at 10 and 15 min following the second contrast injection. A Modified Look-Locker Inversion Recovery (MOLLI) sequence with a 5 (4) 3 acquisition strategy was used with parameters as follows: TE 1.1 ms, TR 2.5 ms FA 35°, FOV 340 × 260 mm, resolution 1.8 mm × 1.8 mm × 8 mm.

Late gadolinium enhancement (LGE) was performed at least 5 min following the second gadolinium bolus injection for stress perfusion imaging, using a phase sensitive inversion recovery sequence (TR 650 ms, TE 4.2 ms, FA 25°, FOV 300-340 mm, resolution 1.8 × 1.3 × 8 mm).

#### Cardiac computed tomography (CT)

##### Calcium scoring

Additionally, subjects with risk factors for MVD underwent coronary artery calcium scoring (CAC) to measure coronary atherosclerotic plaque burden. CAC scoring was performed on a Siemens FLASH CT Scanner (Siemens Healthineers, Munich, Germany) using high-pitch spiral mode. Non-contrast CT acquisition parameters included 120 kV, 80 mAs ref. mAs, and reconstructed slice thickness of 3 mm.

### Image analysis

#### Functional assessment and LGE analysis

CMR images were analyzed by an experienced investigator using QMASS (Medis Medical Imaging Systems, Leiden, the Netherlands) on a Leonardo workstation. Cine images were semi-automatically contoured for each short-axis slice, with total LV mass, end-diastolic volume, end-systolic volume, stroke volume and ejection fraction measured [20]. LGE images were visually assessed for the presence of myocardial infarction or scar by an experienced cardiologist.

#### Perfusion quantification

Perfusion images were reconstructed using non-Cartesian SPIRiT [21], and then aligned with non-rigid registration using the ANTs toolbox [22]. Perfusion images were normalized by the PD images and Bloch simulation was used to convert normalized signal intensity to gadolinium concentration prior to Fermi-function deconvolution [23]. Quantification of first-pass perfusion was performed on a pixel-wise basis using the constrained Fermi function deconvolution method implemented in MATLAB (Mathworks, Natick, Massachusetts, USA) [14]. Global stress and rest perfusion were determined for each subject. MPR was calculated as a ratio of the stress perfusion divided by rest perfusion for each subject.

#### T1 analysis

T1 quantification was performed by manual segmentation of the myocardium with care to avoid partial volume effects with the LV cavity. The partition coefficient of gadolinium, λ, was determined from the slope of the linear fit of the plot of 1/T1 myocardium versus 1/T1 blood. The extracellular volume fraction (ECV) was calculated as λ *(1-Hematocrit). Hematocrit was measured on the day of the CMR study.

#### CT calcium scoring

The CAC studies were analyzed by determining the Agatston score on a per-patient basis using a package on a Carestream PACs system (Carestream Health, Rochester, New York, USA).

### Statistical analysis

Analysis was conducted using SAS, version 9.4 (SAS Institute, Inc., Cary, North Carolina, USA). Continuous normally distributed data are presented as mean ± SD, and were compared using two-sided t-tests. Normality of continuous variables was assessed using the Shapiro-Wilk Test. Non-normally distributed data are presented as median and interquartile range (quartile 1 and quartile3 in brackets) and were compared using the Exact Wilcoxon-Mann-Whitney test. Categorical data are presented as N and percentages and compared using the Fisher Exact Test. For all statistics a *p* < 0.05 was considered statistically significant.

The effects of LV mass, body mass index (BMI), age, and gender on the differences in MPR, and rest and stress myocardial perfusion between the subjects and controls were assessed using PROC GLM to perform linear modelling. The goal of this linear modelling was to determine if the mean perfusion parameters were different between subjects and controls when other covariates were included in the model. The LSMEANS statement was used to determine the adjusted group means for the subjects and controls, and to determine whether they remained statistically different after appropriately adjusting for the other covariates in the model. When appropriate multiple comparisons were adjusted for using the Tukey method.

## Results

### Patient characteristics

A total of 46 subjects with risk factors for MVD and no obstructive CAD, and 20 healthy control subjects were included in the study. The subjects’ and controls’ baseline characteristics are summarized in Table 1. The subjects and controls had similar ages (57.5 ± 11.2 years vs 53.4 ± 11.9 *p* = 0.20). The percentage of female subjects were similar between the groups (*p* = 0.38). The subjects had a higher BMI as compared to the controls (31.3 ± 6.0 vs 24.3 ± 3.6, *p* < 0.001). The study group had a higher incidence of diabetes, hypertension, hyperlipidemia, and smoking than the control subjects. The median calcium score in the study population was 0.6.

**Table 1:** Patient Characteristics

Characteristic	Subjects (*n* = 46)	Healthy Controls (*n* = 20)	*P* value
Age	57.5 ± 11.2	53.4 ± 11.9	0.195
BMI	31.3 ± 6.0	24.3 ± 3.6	< 0.001
Female	34 (74)	12 (60)	0.382
Diabetes	37 (80)	0 (0)	0.048
History of smoking	25 (54)	1 (5)	< 0.001
Hypertension	37 (80)	1 (5)	< 0.001
Hyperlipidemia	40 (87)	1 (5)	< 0.001

Values listed are mean ± SD or n (%). BMI = body mass index

### CMR data analysis

Subjects and controls had a normal mean LV ejection fraction (LVEF) (61.2 ± 6.7% versus 59.7 ± 4.3%, respectively, *p* = 0.26). Native T1 values (988 ± 42 ms subjects vs. 987 ± 35 ms controls, *p* = 0.87), and extracellular volume fraction (ECV) (26.6 ± 2.9subjects vs. 27.1 ± 2.2controls, *p* = 0.44) were similar between groups. LV mass was similar in subjects and controls, 76.3 g [63.6, 94.8] g as compared to 67.8 g [56.9, 88.8] (*p* = 0.1806). These findings are listed in Table 2. No LGE was visualized in any subjects or controls.

**Table 2:** MRI Parameters

Parameter	Subjects (*n* = 46)	Controls (*n* = 20)	*P*-value
Rest Perfusion (ml/g/min)	1.11 [0.99,1.37]	1.11 [0.92,1.23]	0.25
Stress Perfusion (ml/g/min)	2.65 ± 0.62	3.17 ± 0.49	0.002
MPR	2.21 [1.95,2.69]	2.93 [2.763.19]	< 0.001
LVEF (%)	61.2 ± 6.7	59.7 ± 4.3	0.26
LV mass (g)	76.3 [63.6,94.8]	67.8 [56.9,88.8]	0.18
ECV (%)	26.6 ± 2.9	27.1 ± 2.2	0.44
Native T1 (ms)	988 ± 42	987 ± 35	0.87

*ECV* extracellular volume fraction, *LV* left ventricular, *LVEF* left ventricular ejection fraction, *MPR* myocardial perfusion reserve

### Quantitative perfusion analysis

**Fig. 1:**
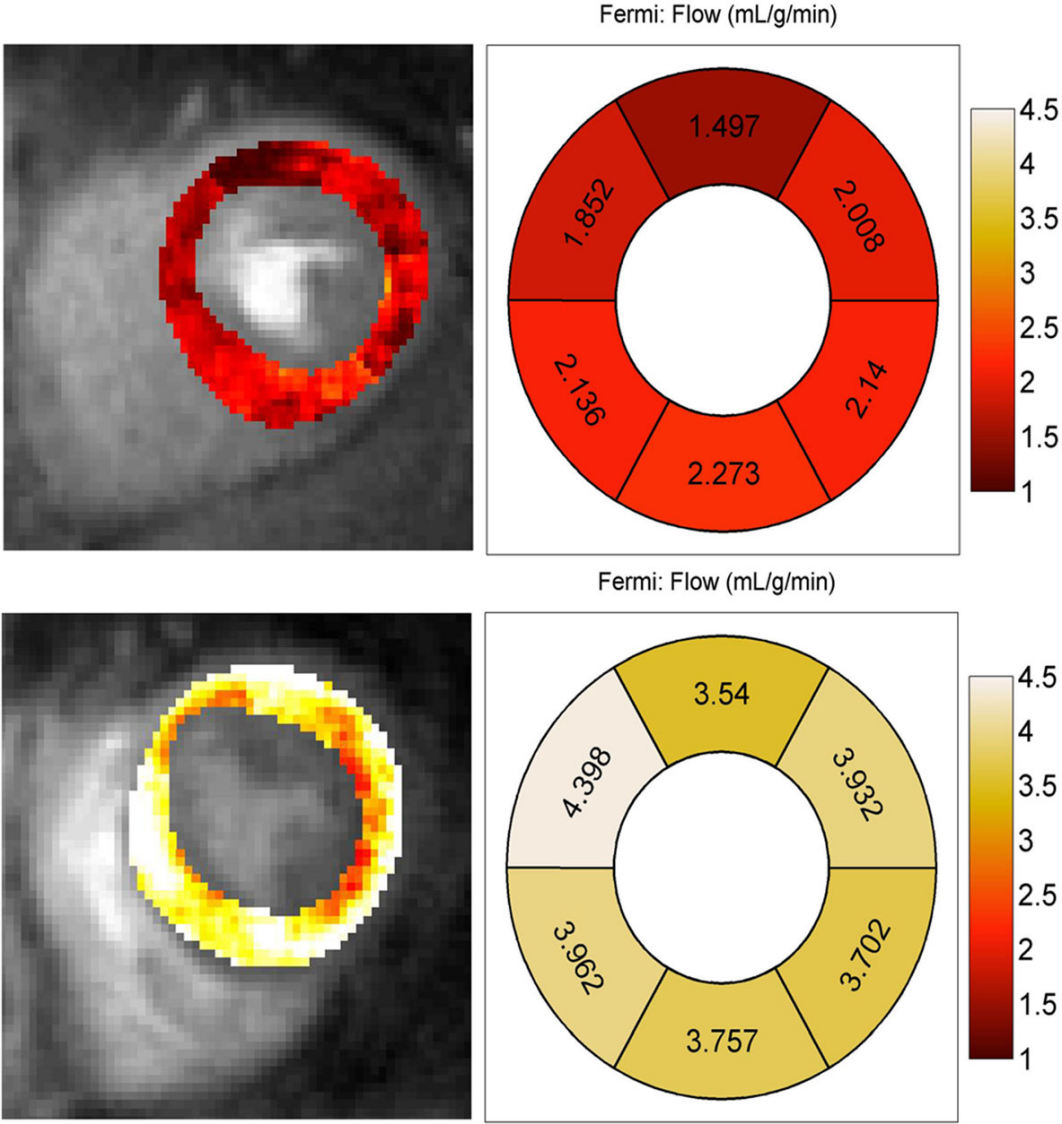
Stress and Rest Absolute Perfusion Quantification. Comparison of absolute stress flow values of mid-ventricular short axis slice. Left column: Pixel based flow map. Top Row: Patient with microvascular disease (MVD). Bottom Row: Control subject

Figure 1 shows pixel-wise quantitative stress perfusion maps and regional absolute stress myocardial blood flow (MBF) per AHA 17 segment model mid-ventricular slice in (a) a subject with risk factors for MVD and (b) a control subject. Median resting MBF was similar in subjects and controls, with values of 1.11 [0.99, 1.37] ml/min/g in subjects versus 1.11 [0.92, 1.23]ml/min/g in controls (*p* = 0.25). Across both groups, female subjects had higher mean resting flow then male subjects, 1.26 ± 0.25 ml/min/g versus 0.93 ± 0.14 ml/min/g, respectively (*p* < 0.001). In linear modelling, age (*p* = 0.27) and BMI (*p* = 0.55), and LV mass (*p* = 0.57) were not significant linear predictors of resting MBF (Additional file 1).

**Fig. 2:**
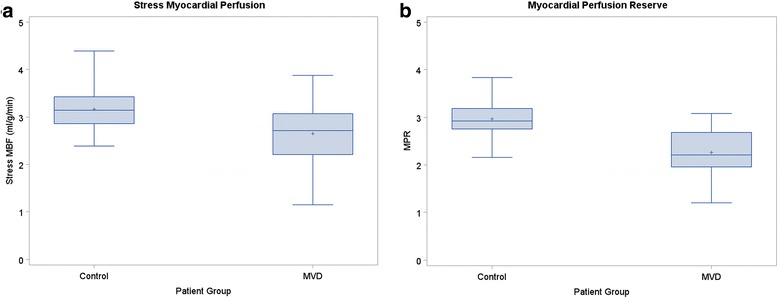
Comparison of Stress myocardial blood flow (MBF) and myocardial perfusion reserve (MPR). Box and whisker plots representing stress MBF (**a**) and MPR (**b**), between study patients and controls. Stress MBF and MPR were lower in study patients by statistically significant values, *p* < 0.002 and *p* < 0.001, respectively. The + sign indicates the mean of each patient group

The findings regarding stress MBF and MPR are presented in Fig. 2. Stress MBF was significantly lower in the subjects 2.65 ± 0.62 ml/min/g, when compared to the mean stress flow in controls, 3.17 ± 0.49 ml/min/g (*p* = 0.002). In the adjusted linear model for stress MBF, the differences in stress MBF between subjects and controls remained significant (*p* = 0.028). The covariates of gender (p < 0.001), and BMI (*p* = 0.002) but not age (*p* = 0.15) were also significantly predictive of stress MBF (Additional file 1).

Median MPR was lower in study subjects versus controls (2.21 [0.73] vs. 2.93 [0.44], p < 0.001). In the adjusted linear model for MPR, the differences in MPR between subjects and controls (p < 0.001) and BMI (*p* = 0.03) were the only significant effects. Covariates of gender (*p* = 0.58) and age (*p* = 0.78) were not significant predictors of MPR (Additional file 1). There was no significant correlation of MPR and stress perfusion with ECV or native T1.

A total of 57% (*N* = 26) of the MVD group subjects had a CAC score < 1 Agatston units, and 13 subjects (28%) had a CAC score > 10 Agatston units. However, there was no difference in median resting MBF between subjects with or without CAC, (1.10 [1.03 1.38] ml/min/g vs 1.14 [0.90,1,36] *p* = 0.50). Similarly, there was no difference in mean stress MBF between subjects when stratified above and below the median CAC score of 0.6 Agatston units, 2.56 ± 0.74 ml/min/g versus 2.72 ± 0.53 ml/min/g (*p* = 0.40). Consequently, median MPR was not significantly different above and below the median CAC score, (2.22 [1.81,2.82] as compared to 2.29 [2.02,2.58] *p* = 0.61).

Per the inclusion criteria, none of the patients had a stenosis greater than 50%. There were 9 subjects (20%) with non-flow limiting stenosis in the 25%-45%. There was no significant difference resting perfusion, stress perfusion, or MPR between those with non-obstructive CAD > 25% as compared to those without a 25% luminal narrowing (*p* = 0.17,*p* = 0.25,*p* = 0.23 respectively).

## Discussion

Using quantitative CMR first pass perfusion imaging, the present study demonstrates that patients who are at high risk for MVD with angina or angina equivalent symptoms with no obstructive CAD have reduced stress MBF and reduced MPR compared with normal controls. This suggests that these patients likely have CMD as a cause for their symptoms. The subjects had an increased LV mass index and BMI, but neither accounted for the reduced MPR. Coronary arteriolar vasomotor dysfunction in response to adenosine is likely a contributing factor, as our group has demonstrated abnormal vasodilatory response of arterioles to graded doses of adenosine in a murine model of MVD [24].

CMR first-pass perfusion imaging has been used primarily in the assessment of perfusion in patients with obstructive CAD, with multiple studies showing high diagnostic accuracy when compared to invasive techniques [13, 25, 26]. In one study, MPR assessed by CMR and PET demonstrated a strong correlation; however, due to differential biases between the techniques absolute MBF was more weakly correlated [27]. When using quantitative coronary angiography as the gold standard, quantitative stress perfusion CMR demonstrates superior performance for detecting obstructive CAD than visual and semi-quantitative methods [26]. Additionally, our group has previously demonstrated that quantitative CMR perfusion imaging detected a larger burden of ischemia in multi-vessel disease as compared to visual analysis [13]. Thus, given the relative efficacy of quantitative assessment of MPR using stress perfusion CMR in obstructive disease, quantitative stress perfusion CMR is important to study in the evaluation of MVD.

This study’s findings extend beyond previous assessments of MPR in those with MVD by qualitative and semi-quantitative perfusion CMR techniques. In a small cohort of patients with normal coronary arteries, Wohrle et al. demonstrated a correlation between CMD and MPR index measured by semi-quantitative analysis [28]. Thomson et al., as part of the WISE study group, examined 118 subjects and demonstrated that women with CMD proven by coronary reactivity testing can be identified with semi-quantitative perfusion CMR [29], which expanded on earlier work with similar results [30]. However, absolute assessment of MPR by CMR has advantages over semi-quantitative methods. Semi-quantitative assessment, as calculated by the ratio of the contrast upslope in the myocardium during stress and rest normalized by the blood pool upslope, is simple to perform but can be biased by signal-saturation in the blood pool due to the high concentrations of gadolinium. Quantitative analysis of myocardial perfusion avoids this bias by curve fitting to the end of the first-pass; however, calculated blood flow values can differ significantly by the specifics of the pulse sequence, limiting direct comparison between studies or across modalities. Calculating MPR indices obviates this latter limitation.

Our data demonstrates that patients at risk for MVD have similar native T1 and ECV to control subjects suggesting that these subjects may not have significant myocardial fibrosis, or sufficient inflammation/edema to cause abnormalities in native T1 or ECV. Furthermore, we saw no correlation between ECV and Native T1 with stress perfusion or MPR. Similar findings were seen in the recent paper from the iPOWER group, which demonstrated no correlation between MVD and fibrosis, as measured by CMR [31].

Notably, while rest and stress flows were higher in females than males, there was no gender difference in MPR. This has also been demonstrated previously in the PET literature [32]. In our study age was not a significant predictor of perfusion parameters. While we saw no significant relationship between CAC score and perfusion reserve, the majority of patients had very low CAC scores, and none of the patients had obstructive CAD.

There are some limitations to the present study. This study investigated patients who were high risk for MVD and had no obstructive CAD by coronary angiography. Due to study enrollment after coronary angiography, invasive CFR and coronary reactivity testing were not acquired at the time of angiography to validate the diagnosis of MVD and to compare with the quantitative CMR findings. The control group had a lower mean BMI than the study group. While the sample sizes of the two groups are different, with the current sample sizes there was > 90% power to detect a difference in stress flow between the groups. Finally, while the sample size of the current study does not allow for detailed subgroup analysis, the findings persisted when corrected for age, gender, BMI, and LV mass. A larger study will be needed to clarify any relationships between subgroups of patients within the MVD group and myocardial perfusion.

## Conclusion

We have demonstrated by quantitative CMR perfusion imaging that in patients at high risk for MVD with angina and no obstructive CAD, there is a reduction in absolute stress myocardial blood flow, and an overall reduction in MPR when compared with normal controls, suggesting that MVD may be a possible cause of symptoms. Further studies with larger sample sizes, comparison to invasive assessment of MVD, and outcome measures are warranted. Future studies of MVD utilizing quantitative CMR perfusion may provide novel insights into MVD pathophysiology and may be helpful in the development of novel therapies.

## Additional file

Additional file 1: Quantitative CMR perfusion imaging identifies reduced flow reserve in microvascular CAD. (DOCX 19 kb)

## Abbreviations

AIF: Arterial input function
BMI: Body mass index
bSSFP: Balanced steady state free precession
CAC: Coronary artery calcium
CAD: Coronary artery disease
CFR: Coronary flow reserve
CMD: Coronary microvascular dysfunction
CMR: Cardiovascular magnetic resonance
CT: Computed tomography
ECV: Extracellular volume
FOV: Field of view
LV: Left ventricle/left ventricular
LVEF: Left ventricular ejection fraction
LVH: Left ventricular hypertrophy
MBF: Myocardial blood flow
MOLLI: Modified Look-Locker Inversion Recovery
MPR: Myocardial perfusion reserve
MVD: Microvascular disease
PD: Proton density
PET: Positron emission tomography
RPP: Rate-pressure product
SR: Saturation recovery
TE: Echo time
TR: Repetition time
