# Supplementary material for: Quantitative cardiovascular magnetic resonance perfusion imaging identifies reduced flow reserve in microvascular coronary artery disease
Source: J Cardiovasc Magn Reson. 2018 Feb 22;20:14. doi: 10.1186/s12968-018-0435-1 (PMC5822618; doi:10.1186/s12968-018-0435-1)
Supplement: Supplementary file 1 — Quantitative CMR perfusion imaging identifies reduced flow reserve in microvascular CAD. (DOCX 19 kb) [file 12968_2018_435_MOESM1_ESM.docx]

**Quantitative CMR perfusion imaging identifies reduced flow reserve in microvascular CAD**

**Supplemental Material**

1) Linear Modelling For Rest Perfusion

| Resting Myocardial Blood Flow Model |
| --- |

The GLM Procedure

Dependent Variable: Rest_MBF Rest_MBF

| **Source** | **DF** | **Sum of Squares** | **Mean Square** | **F Value** | **Pr > F** |
| --- | --- | --- | --- | --- | --- |
| **Model** | 5 | 1.66603552 | 0.33320710 | 6.35 | <.0001 |
| **Error** | 60 | 3.15019907 | 0.05250332 |  |  |
| **Corrected Total** | 65 | 4.81623459 |  |  |  |

| **R-Square** | **Coeff Var** | **Root MSE** | **Rest_MBF Mean** |
| --- | --- | --- | --- |
| 0.345921 | 19.72713 | 0.229136 | 1.161528 |

| **Source** | **DF** | **Type III SS** | **Mean Square** | **F Value** | **Pr > F** |
| --- | --- | --- | --- | --- | --- |
| **MVD** | 1 | 0.09590981 | 0.09590981 | 1.83 | 0.1816 |
| **Gender** | 1 | 0.72432115 | 0.72432115 | 13.80 | 0.0004 |
| **Age** | 1 | 0.06622541 | 0.06622541 | 1.26 | 0.2659 |
| **bmi** | 1 | 0.01881909 | 0.01881909 | 0.36 | 0.5516 |
| **LV_Mass** | 1 | 0.01706959 | 0.01706959 | 0.33 | 0.5707 |

| Resting Myocardial Blood Flow Model |
| --- |

The GLM Procedure

Least Squares Means

Adjustment for Multiple Comparisons: Tukey-Kramer

| **MVD** | **Rest_MBF LSMEAN** | **H0:LSMean1=LSMean2** |
| --- | --- | --- |
|  |  | **Pr > \|t\|** |
| **0** | 1.03203662 | 0.1816 |
| **1** | 1.13541730 |  |

**Conclusion**: In the linear model adjusted for Gender, Age, BMI, and LV mass, MVD Group (controls (0) and subjects (1)) did not have statistically different resting blood flow.

2) Linear Modelling for Stress Perfusion

| Stress Myocardial Blood Flow Model |
| --- |

The GLM Procedure

Dependent Variable: STRESS_MBF STRESS_MBF

| **Source** | **DF** | **Sum of Squares** | **Mean Square** | **F Value** | **Pr > F** |
| --- | --- | --- | --- | --- | --- |
| **Model** | 5 | 12.44105912 | 2.48821182 | 11.00 | <.0001 |
| **Error** | 60 | 13.56678674 | 0.22611311 |  |  |
| **Corrected Total** | 65 | 26.00784586 |  |  |  |

| **R-Square** | **Coeff Var** | **Root MSE** | **STRESS_MBF Mean** |
| --- | --- | --- | --- |
| 0.478358 | 16.93748 | 0.475514 | 2.807464 |

| **Source** | **DF** | **Type III SS** | **Mean Square** | **F Value** | **Pr > F** |
| --- | --- | --- | --- | --- | --- |
| **MVD** | 1 | 1.15357720 | 1.15357720 | 5.10 | 0.0275 |
| **Gender** | 1 | 4.80477706 | 4.80477706 | 21.25 | <.0001 |
| **Age** | 1 | 0.48229194 | 0.48229194 | 2.13 | 0.1494 |
| **bmi** | 1 | 2.27443251 | 2.27443251 | 10.06 | 0.0024 |
| **LV_Mass** | 1 | 0.31973925 | 0.31973925 | 1.41 | 0.2391 |

| Stress Myocardial Blood Flow Model |
| --- |

The GLM Procedure

Least Squares Means

Adjustment for Multiple Comparisons: Tukey-Kramer

| **MVD** | **STRESS_MBF LSMEAN** | **H0:LSMean1=LSMean2** |
| --- | --- | --- |
|  |  | **Pr > \|t\|** |
| **0** | 2.90941693 | 0.0275 |
| **1** | 2.55088222 |  |

**Conclusion**: In the linear model adjusted for Gender, Age, BMI, and LV mass, the stress myocardial blood flow was different for the controls and subjects.

3) Linear Modelling for Myocardial Perfusion Reserve

| Myocardial Perfusion Reserve |
| --- |

The GLM Procedure

Dependent Variable: MPR MPR

| **Source** | **DF** | **Sum of Squares** | **Mean Square** | **F Value** | **Pr > F** |
| --- | --- | --- | --- | --- | --- |
| **Model** | 5 | 8.26409132 | 1.65281826 | 8.98 | <.0001 |
| **Error** | 60 | 11.04357515 | 0.18405959 |  |  |
| **Corrected Total** | 65 | 19.30766647 |  |  |  |

| **R-Square** | **Coeff Var** | **Root MSE** | **MPR Mean** |
| --- | --- | --- | --- |
| 0.428021 | 17.34338 | 0.429022 | 2.473691 |

| **Source** | **DF** | **Type III SS** | **Mean Square** | **F Value** | **Pr > F** |
| --- | --- | --- | --- | --- | --- |
| **MVD** | 1 | 3.12895249 | 3.12895249 | 17.00 | 0.0001 |
| **Gender** | 1 | 0.05593260 | 0.05593260 | 0.30 | 0.5835 |
| **Age** | 1 | 0.01457257 | 0.01457257 | 0.08 | 0.7794 |
| **bmi** | 1 | 0.95526062 | 0.95526062 | 5.19 | 0.0263 |
| **LV_Mass** | 1 | 0.73410369 | 0.73410369 | 3.99 | 0.0504 |

| Myocardial Perfusion Reserve |
| --- |

The GLM Procedure

Least Squares Means

Adjustment for Multiple Comparisons: Tukey-Kramer

| **MVD** | **MPR LSMEAN** | **H0:LSMean1=LSMean2** |
| --- | --- | --- |
|  |  | **Pr > \|t\|** |
| **0** | 2.86927819 | 0.0001 |
| **1** | 2.27879526 |  |

**Conclusion**: In the linear model adjusted for Gender, Age, BMI, and LV mass, the myocardial perfusion reserve was different for the controls and subjects.

**Note**: Type III SS, which are invariant to the order of the effects in the model, are presented. Models are thus adjusted for all of the covariates in the model, not just in sequential order.
